# Effects of low intensity pulsed ultrasound with and without increased cortical porosity on structural bone allograft incorporation

**DOI:** 10.1186/1749-799X-3-20

**Published:** 2008-05-27

**Authors:** Brandon G Santoni, Nicole Ehrhart, A Simon Turner, Donna L Wheeler

**Affiliations:** 1 Department of Mechanical Engineering, School of Biomedical Engineering, Orthopaedic Bioengineering Research Laboratory, Colorado State University, Fort Collins, CO 80523, USA; 2 Department of Clinical Sciences, James L. Voss Veterinary Medical Center, Colorado State University, Fort Collins, CO 80523, USA; 3 BioSolutions Consulting LLC, 385 Coastal View Drive, Webster, NY 14580, USA

## Abstract

**Background:**

Though used for over a century, structural bone allografts suffer from a high rate of mechanical failure due to limited graft revitalization even after extended periods *in vivo*. Novel strategies that aim to improve graft incorporation are lacking but necessary to improve the long-term clinical outcome of patients receiving bone allografts. The current study evaluated the effect of low-intensity pulsed ultrasound (LIPUS), a potent exogenous biophysical stimulus used clinically to accelerate the course of fresh fracture healing, and longitudinal allograft perforations (LAP) as non-invasive therapies to improve revitalization of intercalary allografts in a sheep model.

**Methods:**

Fifteen skeletally-mature ewes were assigned to five experimental groups based on allograft type and treatment: +CTL, -CTL, LIPUS, LAP, LIPUS+LAP. The +CTL animals (n = 3) received a tibial ostectomy with immediate replacement of the resected autologous graft. The -CTL group (n = 3) received fresh frozen ovine tibial allografts. The +CTL and -CTL groups did not receive LAP or LIPUS treatments. The LIPUS treatment group (n = 3), following grafting with fresh frozen ovine tibial allografts, received ultrasound stimulation for 20 minutes/day, 5 days/week, for the duration of the healing period. The LAP treatment group (n = 3) received fresh frozen ovine allografts with 500 μm longitudinal perforations that extended 10 mm into the graft. The LIPUS+LAP treatment group (n = 3) received both LIPUS and LAP interventions. All animals were humanely euthanized four months following graft transplantation for biomechanical and histological analysis.

**Results:**

After four months of healing, daily LIPUS stimulation of the host-allograft junctions, alone or in combination with LAP, resulted in 30% increases in reconstruction stiffness, paralleled by significant increases (p < 0.001) in callus maturity and periosteal bridging across the host/allograft interfaces. Longitudinal perforations extending 10 mm into the proximal and distal endplates filled to varying degrees with new appositional bone and significantly accelerated revitalization of the allografts compared to controls.

**Conclusion:**

The current study has demonstrated in a large animal model the potential of both LIPUS and LAP therapy to improve the degree of allograft incorporation. LAP may provide an option for increasing porosity, and thus potential *in vivo *osseous apposition and revitalization, without adversely affecting the structural integrity of the graft.

## Background

Skeletal healing requires the spatial and temporal orchestration of numerous cell types, growth factors and genes working in unison towards restoring bone's structural integrity and function. Much thought has been devoted to accelerating or augmenting these reparative processes. Biophysical stimulation has been investigated experimentally and clinically as an orthopaedic intervention for several decades and positive results have been reported for fracture healing, [1-3] delayed unions and non-unions,[4,5] and biomaterial osteointegration[6] These interventions include pulsed electromagnetic fields (PEMF),[7] low intensity ultrasound (LIPUS),[1,2] high frequency, low magnitude mechanical stimuli,[8,9] and direct electric current[4] The scientific underpinning for these biophysical approaches is that they serve as exogenous surrogates for the regulatory signals normally arising through skeletal loading which are absent because of sustained trauma.

Since clinical introduction in the 1950s, ultrasound at intensities ranging from 1 to 50 mW/cm^2 ^has been demonstrated to be osteogenic, chondrogenic, and angiogenic, thus accelerating skeletal healing in animal[10,11] and human clinical studies [1-3]*In vitro *cell-culture experiments have shown LIPUS upregulates osteoblastic production of IL-8, basic-FGF, VEGF, TGF-β, alkaline phosphatase, and the non-collagenous bone proteins, [12-15] while concomitantly down-regulating the osteoclastic response[12,13] The documented pro-osteoblastic and anti-osteoclastic findings have prompted recent efforts using LIPUS to mitigate the onset of osteoporosis in patients with spinal cord injuries and for the treatment of osteoporosis in elderly women[16,17] Combining data from *in vitro*, preclinical and clinical studies, enhanced tissue healing (bone and soft tissue) may primarily be due to the stimulatory effects of LIPUS on angiogenesis.

To date, no work has investigated the potential of LIPUS on massive bone allograft incorporation. Though commonplace in clinical orthopaedics, allograft bone incorporates slowly with susceptibility to host/graft non-union, fracture, and fatigue failure resulting in a 50–75% success rate at 10 years [18-20] Improving allograft incorporation is key to a successful reconstruction and improving the long-term clinical outcome. We hypothesized that daily LIPUS stimulation of the host/allograft junctions may accelerate integration of intercalary allografts given the osteostimulatory effects of this signal on adjacent host bone as well as the host bed surrounding the allograft. To date, successful strategies that aim to improve graft incorporation are lacking. Recent animal studies using bone morphogenetic proteins (BMPs) to promote graft integration have had limited success as they have been shown to stimulate resorption more than formation in the early phase[21] Perforating the bony cortex perpendicular to the long axis of the graft has been investigated as a means to improve graft integration, though these modifications have been reported to have mixed results[22,23] When perforation is combined with cortical demineralization, incorporation is greatly enhanced,[24,25] but at the expense of a 40% decrease in the flexural properties of the graft[26] Since allograft repair proceeds initially by increased osteoclastic activity that decreases allograft mass and radiodensity, transplanting a mechanically compromised graft has lead to accelerated failure and abandonment of graft modification by demineralization and perforation[27] The orientation of perforations within the bony cortex and the ensuing effects on graft revitalization has yet to be quantified. Longitudinal perforations (LAP), as opposed to those oriented perpendicular to the long axis of the graft, may provide more direct access for bone remodeling cell infiltration and eliminate the need for cortical demineralization. This form of allograft modification has recently been shown to have minimal effect on allograft strength[28] Furthermore, we hypothesized than combining LAP with daily LIPUS exposure, given the anabolic effects of ultrasound, may further accelerate allograft healing.

Therefore, the goal of this exploratory study was to examine the potential of daily LIPUS stimulation of the host-allograft bone junctions in limbs reconstructed in an ovine tibial intercalary defect with longitudinally perforated or non-perforated fresh frozen allograft. The LIPUS and LAP adjuvant treatments were compared biomechanically and histologically with the natural healing of fresh frozen allograft (-CTL) and autograft (+CTL) in the same model.

## Methods

### Experimental study design

**Figure 1:**
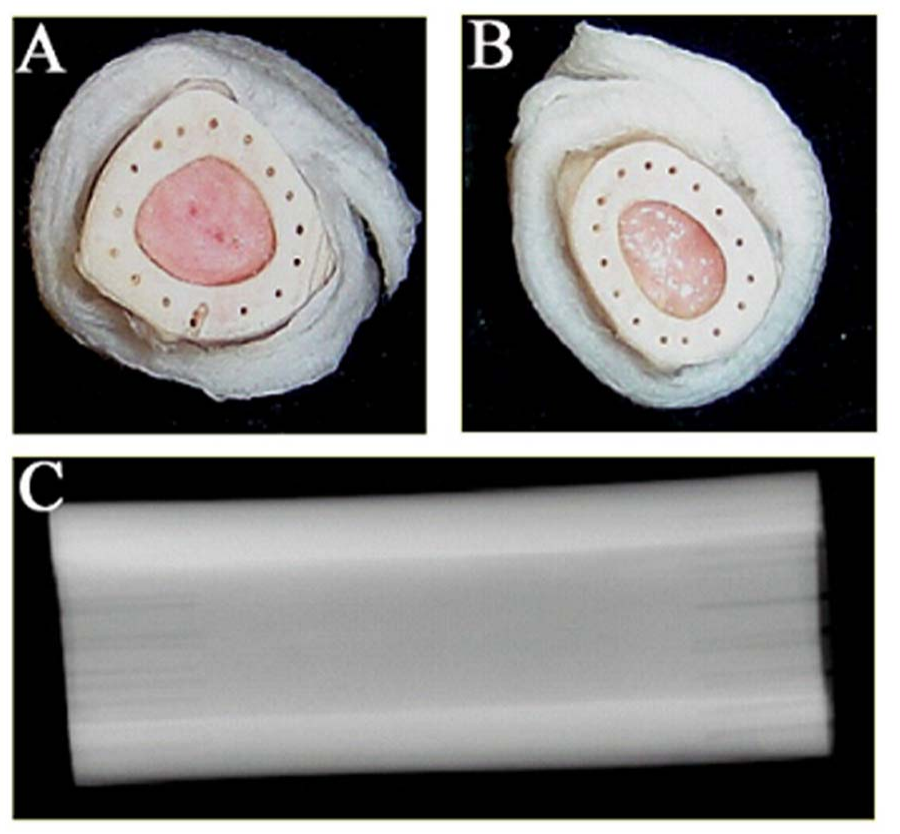
Digital images of perforated 5 cm ovine allografts; (A) proximal and (B) distal perforated end plates illustrate the pattern of 16 conduits that extend 10 mm parallel to the long axis of the allograft; (C) contact radiograph of perforated graft indicating consistency of conduit depth.

This study was approved by the Institutional Animal Care and Use Committee (IACUC #03-297-01) at Colorado State University. Fifteen skeletally-mature ewes were assigned to five experimental groups based on intercalary graft type and treatment: +CTL, -CTL, LIPUS, LAP, LIPUS+LAP. The +CTL animals (n = 3) received a tibial ostectomy with immediate replacement of the resected autologous graft. The -CTL group (n = 3) received fresh frozen ovine tibial allografts. The +CTL and -CTL groups did not receive LAP or LIPUS treatments. The LIPUS treatment group (n = 3), following grafting with fresh frozen ovine tibial allografts, received low-intensity pulsed ultrasound for 20 minutes/day, 5 days/week, for the duration of the healing period. The LAP treatment group (n = 3) received fresh frozen ovine allografts with 500 μm longitudinal perforations that extended 10 mm into the graft (Fig. 1). The LIPUS+LAP treatment group (n = 3) received both LIPUS and LAP interventions. All animals were humanely euthanized four months following graft transplantation for biomechanical and histological analysis.

### Preparation of grafts

Tibial allografts were aseptically harvested from twelve skeletally mature, female sheep. A separate donor was used for each experimental allograft. In a sterile surgical suite, the diaphyseal region of each tibia was palpated and a small medial incision was made overlying the midshaft of the tibia. A 5 cm diaphyseal ostectomy was created with a standard oscillating bone saw. Those grafts assigned to the -CTL and LIPUS groups were immediately wrapped in sterile saline solution soaked gauze and frozen to -80°C. Following ostectomy, the LAP and LIPUS+LAP grafts were placed in 20 cc of 0.9% sodium chloride (NaCl) solution containing polymyxin B sulfate (500,000 units/l), neomycin (1 gram), and ampicillin (3 GM) saline/antibiotic solution. These grafts received sixteen cortical perforations along the longitudinal axis of the graft to a depth of 10 mm in sterile saline using a 500 μm diameter micro-drill bit[28] Perforated grafts were rinsed with saline/antibiotic solution, wrapped in sterile gauze, placed in sealed plastic bags and frozen for at least 4 weeks at -80°C.

### Animal model & surgical procedure

Prior to transplantation, the 5 cm bone segments were debrided of any remaining soft tissues and thawed in warm saline/antibiotic solution. The sheep were prepared for tibial ostectomy using standard aseptic techniques under general anesthesia. Anesthesia was induced with intravenous (IV) ketamine (2.2 mg/kg) and diazepam (0.1 mg/kg) and maintained by isofluorane and oxygen inhalation after endotracheal intubation. Prophylactic cephazolin antibiotic (1 g IV) was given at induction and at the end of surgery. With the sheep in dorsal recumbancy, a 12-cm skin incision was created from the left knee joint to the tarsocrural joint to expose the surgical site. Following ostectomy of a 5 cm mid-diaphyseal osteoperiosteal segment, the distal bone segment was reamed with 6 and 8 mm reamers until an 8-mm diameter nail could be accommodated. The thawed allograft was then inserted and aligned so as to maximize the degree of proximal and distal congruency. An 8.0 mm diameter intramedullary nail (Innovative Animal Products, Rochester, MN) was inserted antegrade using an entry portal created in the craniomedial aspect of the tibial plateau and stabilized with 4.5 mm interlocking screws proximally and distally (Synthes, Paoli, PA). The muscle fascia and subcutaneous tissues were closed with 2-0 absorbable synthetic suture material, the skin edges apposed with 2-0 nylon and a soft-padded bandage placed for 5–7 days. Lateral radiographs were taken postoperatively and at four months following euthanasia. Transdermal fentanyl patches (150 μg/hr) and oral phenylbutazone (1 g once daily) were used prior to surgery and for 72 hours after surgery to minimize pain and inflammation. Sheep were kept in pens for the length of the study to limit activity and examined twice daily for signs of pain and ability to bear weight.

### LIPUS signal & duration of treatment

Animals in the LIPUS and LIPUS+LAP groups were prepped and fitted with two ultrasound transducers within 72 hours following surgery. Ultrasound gel was applied to the experimental limb on the cranial/medial aspect the tibia at both the proximal and distal graft-host junctions and the transducers were affixed using a custom retaining and alignment strap. The 1.5 MHz ultrasound signal generated by the Exogen 2000+ SAFHS device (Smith & Nephew, Memphis, TN) consisted of a 200 μs burst sine wave repeating at a frequency of 1.0 kHz at a signal intensity of 30 mW/cm^2^. Ultrasound exposure was 20 minutes/day for 5 days/week for the four-month study period.

### Biomechanical testing

Following euthanasia, the tibiae were dissected and the surgical hardware removed without disturbing the callus. The experimental and intact contralateral control (iCTL) tibiae were cut to a standard gauge length. The proximal and distal ends of the tibia were potted in specially designed boxes using high strength potting resin (Dynacast, Kindt-Collins Co, Cleveland, OH). Within 12 hours of death, the specimens were mechanically tested in torsion at a rate of 12 degree/sec until failure[29] Right limbs were torqued clockwise and left limbs were torqued counterclockwise to maintain external rotational moments for all tibiae. Digital photographs of the specimens prior to and following failure were taken to document gross appearance, failure mode, fracture pattern, and failure location. Ultimate torque at failure (N-mm) and torsional stiffness (N-mm/deg) were derived from plots of torque and angular displacement data and normalized to the value of the iCTL to minimize the inherent variability in torsional properties between animals.

### Tissue preparation

Following biomechanical testing, transverse cuts were made 2 cm proximal and distal to allograft span. The segment was then sectioned in the sagittal plane, creating medial and lateral tibial halves. Medial halves were sectioned into proximal and distal sections containing approximately 2.5 cm of allograft plus 2 cm of host bone and processed for undecalcified histology. The lateral halves were sectioned by two transverse cuts creating three tissue specimens: two 3.25 × 1 cm proximal/distal sections (allograft + host bone) and a 2.5 × 1 cm middle section (allograft only). The tissue samples from the lateral half were placed in 10% neutral formalin for fixation and processed for decalcified histology.

### Decalcified histological analysis

Following fixation in 10% neutral formalin, those specimens from the lateral half of the reconstructed limb were delcalcified in a 12.5% HCl-EDTA solution, dehydrated in graded solutions of ETOH (75%–100%), and cleared with xylene. The specimens were then processed using standard paraffin histology techniques. Two, 5 μm sections were cut from the specimen block and stained with Hematoxylin and Eosin (H&E) for semi-quantitative evaluation of the degree of allograft revitalization, vascular infiltration, fibrous tissue development and evidence of an elicited immune response according to a scoring system developed in our laboratory (Table 1). Evaluated parameters included the quality and degree of osseous bridging across the host/graft junctions, allograft vascularity, the presence/absence of a chronic immune response, and the degree of callus formation and maturation. Metabolic activity and remodeling in the host and graft were scored (Ob/Oc continuum) based on the presence of osteoblasts (Ob) and newly deposited bone tissue relative to osteoclasts (Oc) and scalloped surfaces.

**Table 1:** Decalcified Histology Scoring System Designed to Quantify Allograft Healing

		CONNECTIVITY		Ob/Oc CONTINUUM	GRAFT REVITALIZATION	
			
Score	Host-Allograft Cortical Bridging	Callus Tissue Type/Host-Graft Direct Interface	Callus (% of c.t.)	Host & Graft	Graft Vascularity	Live Cells In Graft
0	No bridging	Fibrous/pseudoarthrosis	None	No resorption	None	No
1	BC on 1 side	Cartilage	50%	Extensive resorption/No Ob	Some	Yes
2	BC on 2 sides	Endochondral ossification	100%	Some Resorption/No Ob	Moderate	
3	BC and cortex on 1 side	Bone	150%	Extensive Resorption/Some Ob	Prolific	
4	BC and cortex on 2 sides		200%	Some Resorption/Some Ob		
5			> 200%	Some Resorption/Extensive OB		
6				Extensive Resorption/Extensive OB		

Legend: BC = bridging callus; Ob = osteoblast; Oc = osteoclast; callus area thickness is expressed as percent (%) of cortical thickness (c.t.)

### Undecalcified histological analysis

**Figure 2:**
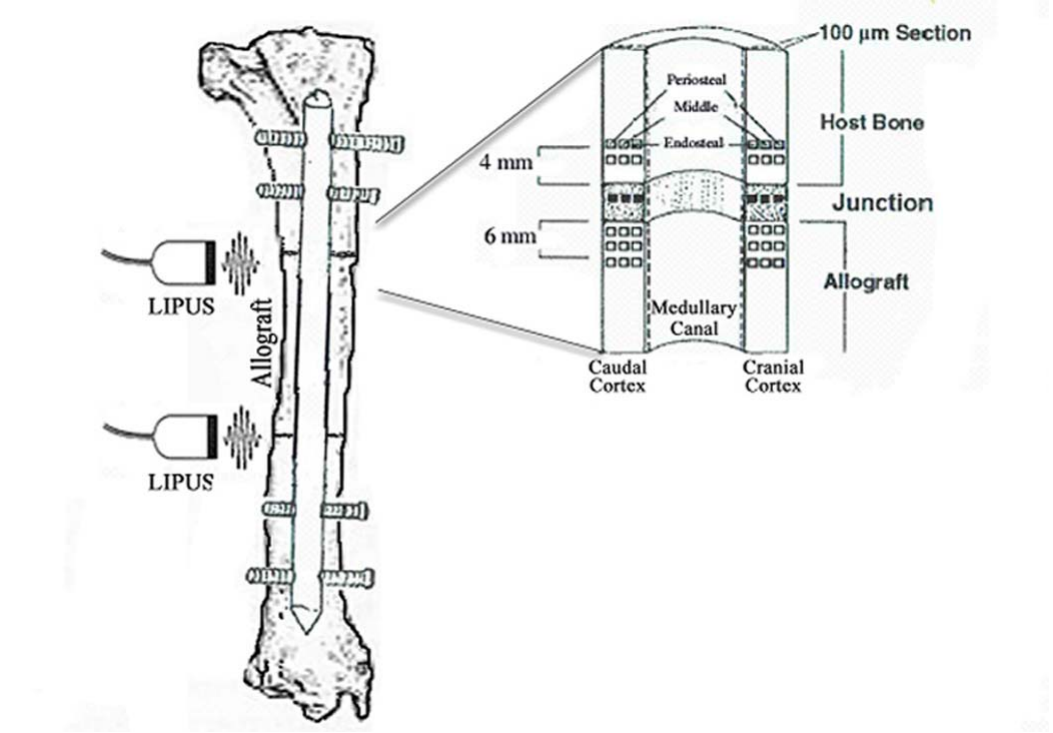
(left) Illustration of tibial defect filled with a 5 cm intercalary allograft and stabilized with a static, interlocking IM nail; these junctions were stimulated daily with LIPUS and/or modified with LAP; proximal (right) and distal (not shown) host graft junctions containing 2 cm of host bone and 2.5 cm of allograft were transected in the sagittal plane allowing for quantification of mineral apposition rate (MAR) in the cranial and caudal cortices as well as the interface regions; regions of interest (ROIs) evaluated were designated as periosteal, middle, and endosteal in the graft, host, and interface from which global averages were derived (animal treated as experimental unit); up to 10 fluorescent images were taken to evaluate MAR in the cranial and caudal callus regions.

Tissues from the medial aspect of the tibia were fixed and dehydrated in graded solutions of ETOH (70%, 95%, and 100%) and xylene over the course of approximately 5 weeks. The samples were infiltrated with a series of solutions containing methyl methacrylate, dibutyl phthalate, and benzoyl peroxide. The final methyl methacrylate solution was polymerized into a hardened plastic block and kept in the dark until completion of dynamic analysis (Fig. 2). One 300 μm section was taken from each specimen block in the sagittal plane using an Exakt diamond blade bone saw (Exakt Technologies, Oklahoma, OK) and subsequently ground to 100 μm thickness using an Exakt microgrinder. The sections remained unstained until after dynamic histomorphometric analyses, and then were subsequently stained with Sanderson's rapid bone stain (Surgipath, Richmond, IL) and acid fuchsin.

High-resolution digital images taken at 20× magnification were acquired for unstained (fluorescent) sections using an Image Pro Imaging system (Media Cybernetics, Silver Spring, MD), a Nikon E800 microscope (AG Heinze, Lake Forest, CA) and Spot digital camera (Diagnostic Instruments, Sterling Heights, MI). The fluorescent images were used to calculate mineral apposition rate (MAR), defined as the average distance between the calcein and tetracycline fluorescent markers divided by the time between label administration, and penetration depth of new bone into allograft tissue in accordance with the standardized system proposed by the American Society for Bone and Mineral Research (ASBMR)[30] Static parameters measured on the stained sectioned included percent of allograft resorbed, calculated as the ratio of scalloped allograft surface relative to total allograft surface, and callus area. All static measurements were made on composite images at 2× magnification. Animal was treated as the experimental unit for statistical analyses of the dynamic and static parameters (n = 3 per treatment group).

### Statistical analysis

Treatment effects for al continuous response variables were evaluated using a one-way ANOVA followed by a Dunnett's post hoc multiple comparison procedure with the allograft only treatment group (-CTL) serving as the control. Decalcified histological parameters were compared with a non-parametric Kruskal-Wallis test. All statistical analyses were conducted using SAS statistical software (SAS Institute, Cary, NC) at a significance level of 0.05.

## Results

### Torsional biomechanics

After 4 months of healing, radiolucent lines were observed at the proximal graft-host junctions in all treatment groups. However, 8 of the 15 distal graft-host junctions (53.3%) were radiographically united. All +CTL animals were united at the distal junction after 4 months yet none of the -CTL were either radiographically or grossly united. Biomechanical failures of all reconstructions occurred at the proximal host/graft junction. LIPUS, LAP, and LIPUS+LAP treated limbs exhibited increased ultimate torque and stiffness compared to -CTL, yet the differences were not statistically significant (Table 2). On average, the +CTL tibiae exhibited structural properties equivalent to intact tibia.

**Table 2:** Results from Torsional Biomechanical Testing

	Structural Properties			
	
	Ultimate Torque (N-mm)		Stiffness (N-mm/deg)	
	
Treatment	%iCTL ± SEM	p-value	%iCTL ± SEM	p-value
(-)CTL	27.80 ± 3.83	-------	24.61 ± 7.18	-------
LIPUS	55.40 ± 18.61	p = 0.229	57.00 ± 13.95	p = 0.163
LAP	41.80 ± 11.65	p = 0.493	51.47 ± 13.06	p = 0.192
LIPUS+LAP	53.40 ± 11.51	p = 0.245	65.03 ± 19.75	p = 0.063
(+)CTL	111.20 ± 19.46	p = 0.002	128.67 ± 5.32	p < 0.001

### Decalcified histology

**Figure 3:**
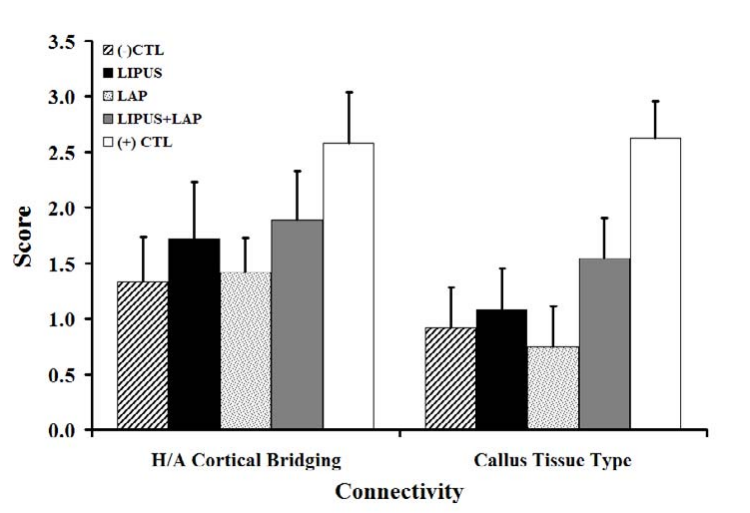
Decalcified connectivity scores. Results presented as Mean ± SEM where the mean was compiled by averaging scores from the proximal, middle and distal slides; connectivity scores were on average higher in the +CTL group relative to all other experimental treatments and the -CTL, which, at four months, elicited a minimal level of osseous bridging; LIPUS treatment appeared to improved osseous bridging and the degree of ossified tissue in the callus; a similar observation was made with the LAP treatment, though the degree of ossified tissue within the callus was smaller; combination treatment seemed to improve direct healing relative to all other treatment groups excluding the +CTL; LEGEND: H = host; A = allo/autograft. All statistical comparisons relative to the -CTL were not significant (p > 0.05).

Connectivity scores (Fig. 3) quantified the degree of osseous bridging between the host and allograft as well as the tissue composition of the periosteal callus. The +CTL exhibited the greatest periosteal bridging and direct cortical-cortical bridging between the host and graft. Conversely, the -CTL demonstrated the poorest degree of osseous bridging relative to all other treatment groups. LIPUS treated junctions had increased periosteal bridging with greater composition of mineralized tissue in the callus mass, while the LAP and -CTL treatments exhibited greater amounts of fibrous and cartilaginous tissues. Surprisingly the LAP treatment group had callus thickness comparable to the LIPUS treated limbs yet the composition of the callus contained less osseous tissue. The combination therapy (LIPUS+LAP) demonstrated marginal improvement in direct healing over the other experimental treatment groups excluding autologous grafts.

Graft revitalization in the experimental treatment groups proceeded periosteally from the highly vascularized and active periosteal callus as evidenced by stained osteocytes residing in this general area. Osteocytes were also evident in grafts adjacent to interface regions that had progressed to a higher level of osseous bridging. On average, the allografted tissue was more viable in the LIPUS and LIPUS+LAP treatment groups relative to the -CTL, though this difference was not significant (p > 0.05). Host tissue adjacent to bone allograft in all treatment groups was highly active as evidenced by high levels of osteoclastic resorption balanced by extensive new bone development (scoring range from Table 1: 4.21–4.75, p > 0.05 for all comparisons) while grafted tissues exhibited more osteoclastic resorption with less new bone development (scoring range from Table 1: 2.97–3.67). Though was especially evident in the -CTL group, no statistical comparisons were significant at the 0.05 level. No inflammatory cells were observed adjacent to the grafts in any treatment group.

### Undecalcified histology

**Figure 4:**
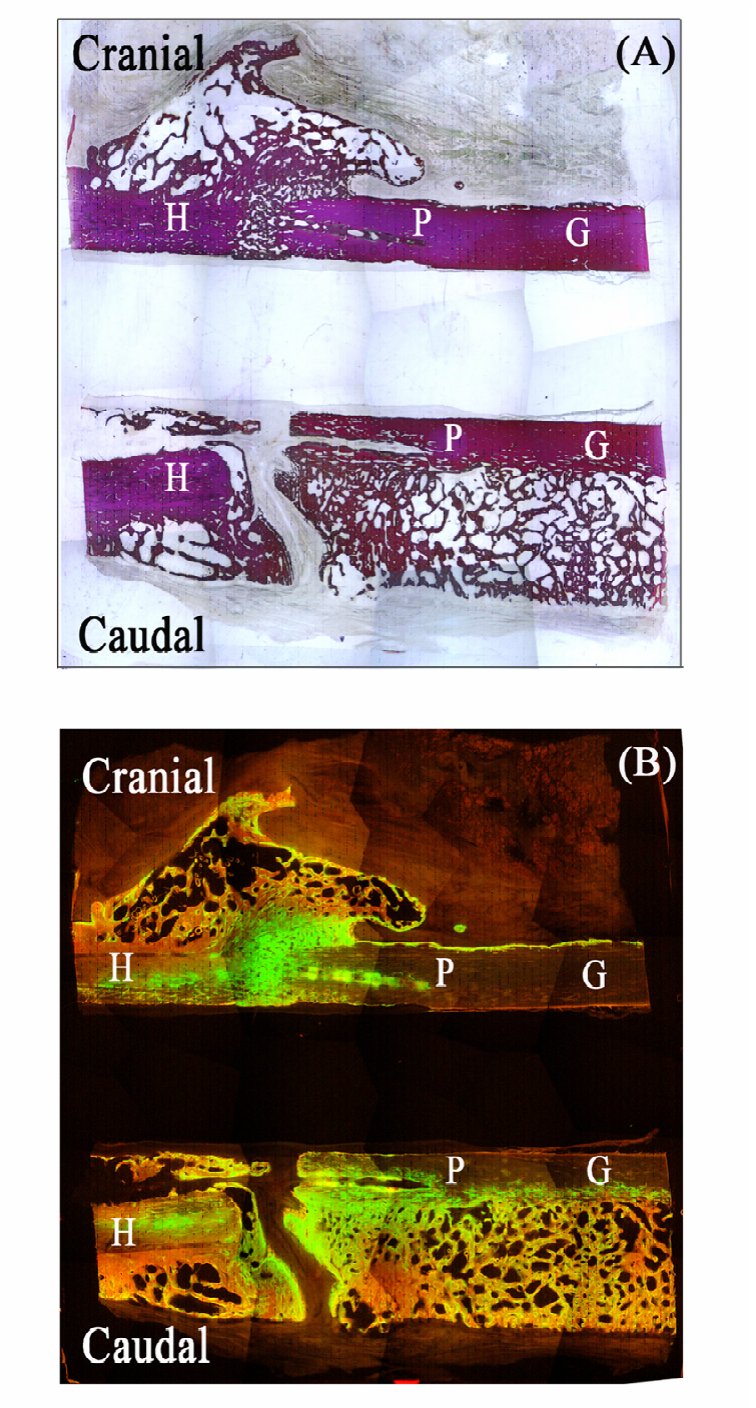
(A) Undecalcified static image (2×) of distal host (H)/graft (G) junction in the LAP treatment group illustrating perforations in the cranial and caudal cortices that have filled to varying degrees with new appositional bone. Cranially (Cr) in (A), the perforation is almost entirely filled with new bone and extends the entire length of the conduit. (B) Dynamic histomorphometric whole specimen composite of (A) (2×) illustrating extensive uptake of both calcein and tetracycline Fluorochrome labels that extend the length of the 10 mm conduits in the cranial and caudal cortices.

**Figure 5:**
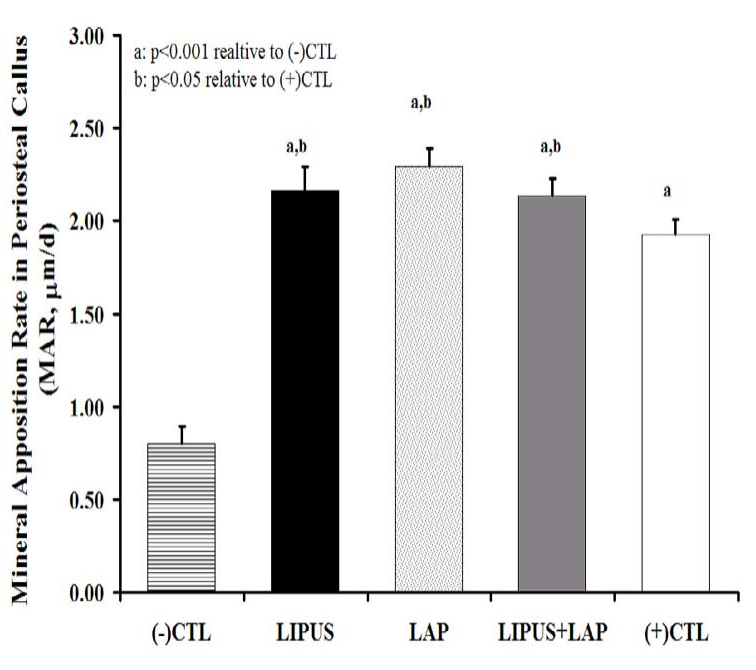
Mineral apposition rates (MARs) quantified in the periosteal callus adjacent to the host/graft junctions. Significant increases in mineral deposition were noted for all treatment groups including the +CTL relative to the -CTL (p < 0.001). MAR in callus exposed to LIPUS and/or adjacent to perforated grafts was also significantly greater than MAR in callus adjacent to autograft (p < 0.05).

Perforations present in the LAP and LIPUS+LAP undecalcified slides filled to varying degrees with immature woven bone after 4 months of healing. Animals within both groups exhibited perforations that were entirely filled with new immature tissue while some animals within those same groups had bone regeneration isolated to the inner surface of the longitudinal perforation. In either case, the appositional bone extended the entire length of the 10 mm perforation in LAP and LIPUS+LAP treatment groups (Fig. 4). Mineralizing tissue extended the entire length of the conduits resulting in graft MAR that was significantly greater in these two treatment groups relative to the -CTL (p < 0.001). LIPUS therapy alone also significantly increased allograft MAR (p < 0.001), though activity was localized at the periphery of the graft rather than mid-cortex as in LAP treated grafts. MAR in the callus was always larger than in the host or allograft for all treatment groups (Figure 5, Table 3). Limbs treated with LIPUS, LAP and LIPUS+LAP had 3-fold greater MAR in the periosteal callus than in -CTL allografts (p < 0.001).

**Table 3:** Summary of MAR in Host, Graft and Interface Regions of Interest

		Mineral Apposition Rate (MAR), mm/d		
		
		Host	Graft	Interface
				
reatment	Cortex	Mean ± SEM	Mean ± SEM	Mean ± SEM
(-)CTL	Cranial	0.242 ± 0.102	0.003 ± 0.004	0.533 ± 0.028
	Caudal	0.248 ± 0.084	0.043 ± 0.025	0.52 ± 0.015
LIPUS	Cranial	1.306 ± 0.197^a^	0.364 ± 0.142^a^	2.058 ± 0.046^a^
	Caudal	1.055 ± 0.214^a^	0.520 ± 0.153^a^	2.580 ± 0.033^a^
LAP	Cranial	0.897 ± 0.162^a^	0.496 ± 0.157^a^	2.543 ± 0.194^a^
	Caudal	0.965 ± 0.18^a^	0.182 ± 0.099^a^	1.608 ± .0.034^a^
LIPUS+LAP	Cranial	0.640 ± 0.142^b^	0.768 ± 0.216^a^	1.597 ± 0.037^a^
	Caudal	0.557 ± 0.146^b^	0.332 ± 0.142^a^	1.964 ± 0.07^a^
(+)CTL	Cranial	0.739 ± 0.15^a^	0.696 ± 0.157^a^	2.243 ± 0.101^a^
	Caudal	0.926 ± 0.176^a^	0.576 ± 0.15^a^	1.932 ± 0.057^a^

a, p < 0.001 relative to (-)CTL.

b, p < 0.010 relative to (-)CTL.

NOTE: there were no significant differences (p > 0.05) for MAR in any region between LIPUS, LAP. or LIPUS+LAP treatment groups.

Qualitatively, distal host/graft junctions progressed to a higher degree of cortical union relative to the proximal junctions within the same animal. Distal periosteal callus was more uniform and continuous, though smaller in total area. Proximal host/graft junctions were characterized by fibrovascular tissue development that tended to isolate the graft from apposing callus impeding union, especially in the -CTL. Scalloped bone surfaces indicative of osteoclastic activity were noted on all allograft-treated groups, though a trend towards decreased activity in the LIPUS group relative to the -CTL was noted. Specifically, on average, 19.59 ± 12.34% of the allograft surface displayed evidence of osteoclastic scalloping on the cranial and caudal surfaces of allografts in the -CTL group, whereas only 13.16 ± 6.09% of the cranial and caudal surfaces in the LIPUS treated allografts contained scalloped areas (p = 0.223). Furthermore, the trend of decreased osteoclastic resorption was more pronounced on the cranial aspect of the graft (i.e. in the direct path of ultrasonic irradiation) on those limbs exposed to the ultrasonic signal (p = 0.156).

## Discussion

The goal of the current study was to elucidate the effects of LIPUS and/or LAP on the incorporation of 5 cm intercalary allografts in an ovine model. Allograft bone is used as an operative treatment option for failed total joint replacements,[31,32] bone tumors,[33,34] and joint diseases[35] since they provide adequate structural support, display mechanical properties similar to the diseased/damaged tissue, and act as an osteoconductor for neovascularization and osteogenic host cells. Although controversy exists as to whether metal implants or osteoarticular allografts are best suited for the treatment of lesions when a joint must be resected,[36,37] intercalary segmental allografts are an accepted treatment option in cases not involving a joint. Though intercalary allografts are easy to insert and stabilize and are associated with relatively lower complication rates than osteochondral allografts, these grafts still suffer from non-union and fracture due to limited and superficial revitalization.[38,39] The results of this study confirm our initial hypotheses and indicate that LAP and/or LIPUS may play a role in improving allograft integration.

Stevenson and Horowitz[40] defined successful cortical bone allograft incorporation as concurrent revascularization following resorption and substitution with host bone without the loss of strength. The initial resorptive phase lasts as long as six months during which graft density decreases by 40%[41] Therefore, therapeutic intervention employed to promote graft healing should not compromise structural properties as has been the case with transversely perforated and demineralized allografts. Though Lewandrowski and coworkers[24,25] have reported promising results using these forms of modification together, the flexural properties of transversely perforated and demineralized grafts were reduced by 40%[26] Premature failure of these grafts *in vivo *has led to abandonment this method of graft modification[27] Delloye et al[22] recently reported complete osseous filling of 1 mm transverse perforations in an ovine model after six months of healing suggesting that perforations alone may have beneficial effects on graft integration without additional demineralization. However, a number of authors have reported negative or negligible beneficial effects of radial perforations alone[23,42] Given these mixed results, conduits extending parallel to the long axis of the graft provide an attractive and as yet uninvestigated alternative.

In the current study, longitudinally-oriented perforations transformed the allograft into an osteoconductive scaffold that promoted osseous revitalization through direct interface with host bone without adversely affecting the structural integrity of the graft. Concomitant mechanical studies confirmed a lack of deleterious effect of conduit presence in both uniaxial and diametral compressions tests as well as finite element analyses[28] The newly formed tissue observed within these longitudinal conduits was thought to have occurred via creeping substitution of reparative tissue from the host/graft interface into the canals. Recent reports have indicated that despite terminal sterilization, processed allografts still maintain significant amounts of osteoinductive proteins[43] Perforating the dense cortical matrix may have exposed an even greater amount of these proteins thus promoting bone apposition within the conduits. To the authors' knowledge, this is the first study to elucidate the structural and biological effects of perforation orientation within massive cortical allografts. Enhanced biologic incorporation of the graft was shown to be independent of cortical demineralization.

Historically, the majority of research with regard to LIPUS relates to fresh fracture healing. Pioneering work by Duarte[44] reported that low-intensity ultrasound accelerated cortical bridging across the site of a fibular osteotomy in rabbits. Work by Pilla et al[45] demonstrated that brief periods of pulsed ultrasound accelerated the recovery of torsional strength and stiffness in rabbit mid-shaft tibial osteotomies and reported biomechanical stability in LIPUS treated limbs in half the time of untreated bones. Additional *in vivo *studies[10,46] suggested that LIPUS acts on the cellular reactions involved in inflammation, angiogenesis, chondrogenesis, intramembranous ossification, endochondral ossification, and bone remodeling. In essence, ultrasound provides an optimal biological and biophysical environment promoting skeletal maintenance and repair. Similar healing effects were found for allograft incorporation as with fresh fracture healing.

Daily ultrasound stimulation of the proximal and distal host/allograft junctions increased torque to failure (27%) and torsional stiffness (33%) of the reconstructed limbs relative to -CTL. Though not statistically significant, the clinical significance of 30–40% increases in reconstruction stiffness following adjuvant LIPUS therapy cannot be understated and warrants further investigation. Future studies with larger sample sizes (n = 8, power = 0.77 versus n = 3, power = 0.54) may substantiate our findings statistically. The increase in structural properties of the reconstructed limbs correlated significantly with three times greater MAR in the periosteal callus. LIPUS appeared to increase periosteal callus size and endochondral ossification, promoting maturation of callus to a dense osseous bridge between the graft and host bone. These findings agree with documented evidence that ultrasound upregulates the biological factors associated with callus formation and maturation, ultimately translating to a more biomechanically competent reconstruction[45] Ultrasound stimulation promoted enhanced periosteal revitalization of the graft compared to -CTL and suggests that even when used as a stand-alone therapy, LIPUS may promote more extensive graft integration and, ultimately, mechanical stability. Our preliminary findings suggest the biologic effects of LIPUS on healing of fresh fractures and non-unions are similar to the mechanisms promoting revitalization of massive allografts.

In the current study, LIPUS administration was unidirectional and stimulated only a small area of the host/graft interface (3.88 cm^2^). Future studies may utilize multiple transducers that deliver separate signals simultaneously to a larger area of the host/graft junctions thereby expanding osteostimulatory effects leading to more uniform callus stimulation and mechanical integrity. Recently, a new mode of LIPUS administration has been developed which employs an internal, intraosseous transducer[11] that minimizes signal attenuation by soft tissues. Though more invasive, this form of administration may expand the application of LIPUS to skeletal sites with more soft-tissue coverage such as femoral allograft reconstructions.

Healing at the host/graft interface for all allograft-treated groups was affected by congruency of the mating surfaces. For all allograft treatments, the proximal host/graft junctions were characterized by fibrovascular tissue development and lack of cortico-cortical union, which was most likely the result of poor cortical-cortical congruency at this junction. The proximal interface had poorer congruency due to the natural curvature of the bone and the mismatch between the larger internal diameter of the tibia and smaller IM nail diameter leading to variability in mating cortices. This, in combination with a lack of direct compression across the interfaces afforded by the IM nail, may have allowed for excessive micromotion, promoting greater immature non-osseous, disconnected callus formation at the interface. This finding may suggest that a 5 cm intercalary defect is too large a defect in this skeletal site. To improve interface congruency an ostectomy either more distal in the tibia or smaller in size should be considered. Compression plate fixation of the intercalary allograft may also provide a mechanical environment more conducive to repair than the IM nail fixation. Nevertheless, new bone was identified on the inner surface of the proximal perforations for the LAP treatment groups despite a lack of cortical bridging proving the osteostimulatory effects of LAP and LIPUS. Further graft revitalization may have been seen with adequate stabilization and better congruency at the graft-host interface.

To the author's knowledge this is the second study that has evaluated the effect of a biophysical stimulus on allograft healing. In the absence of postoperative chemotherapy, Capanna et al[47] reported significant reductions in time to healing in 25 patients treated with PEMF stimulus following tumor resection and limb reconstruction with intercalary or osteochondral allografts. Though the LIPUS and PEMF signals are different in nature, their pro-osteoblastic effects are quite similar[12,13,48] One potential advantage of LIPUS is its reported anti-osteoclastic effects,[12,13] which may have important ramifications to allograft healing. Attenuating osteoclastic activity while upregulating the osteogenic phenotype, especially during the first few months of healing, may maintain graft density and integrity, promoting a better clinical outcome. This is the first *in vivo *study to report decreased osteoclastic response following LIPUS therapy as demonstrated by decreased evidence of osteoclastic scalloping on the allografts exposed to LIPUS. However, this study did not examine the effects of LIPUS on malignant cell types that may remain in the skeletal site following tumor resection. Although Campana et al[47] observed no difference in local tumor recurrence rates in patients receiving PEMF stimulation, future studies are warranted to demonstrate a similar effect following LIPUS therapy.

After four months *in vivo*, combined LIPUS and LAP therapy resulted in 25.7 and 40.4% increases in ultimate torque to failure and stiffness, respectively, relative to untreated allograft reconstructions (-CTL). These gross improvements in structural integrity were paralleled by increases in callus maturity, the extent of callus bridging and the quality of the host/graft interface as measured histologically. The extent to which the longitudinal perforations filled with new bone did not appear to be dependant on exposure to the LIPUS signal thus failing to confirm our initial hypothesis that combination therapy may be significantly synergistic. This finding may be attributed to the acoustic properties of bone, which has a high absorption coefficient, a high acoustic impedance and a capacity to propagate shear waves[16] Resultantly, only 12% of the incident ultrasonic energy was retained in the bone tissue after 1 mm of propagation, a limitation that prevents direct interaction between LIPUS and LAP. Therefore, even if multiple transducers were used, it seems unlikely that the combined therapies would improve the quality and extent of new bone development within the perforations.

## Conclusion

In conclusion, this study has demonstrated in a large animal model the potential of both LIPUS and LAP therapy to improve the degree of allograft incorporation. Future work is warranted to confirm these results in a model with better congruency and stability between the host and graft and to investigate the effects of LIPUS on neoplastic cells.

## Abbreviations

LIPUS: low intensity pulsed ultrasound; LAP: longitudinal allograft perforation; IL-8: interleukin-8; Basic-FGF: fibroblast growth factor; VEGF: vascular endothelial growth factor; TGF-beta: transforming growth factor-beta; BMP: bone morphogenetic protein; SAFHS: sonic accelerating fracture healing system; MAR: mineral apposition rate.

## Competing interests

The authors declare that they have no competing interests.

## Authors' contributions

BGS study conception and design, procurement of funding, acquisition of data, analysis and interpretation of data, drafting of manuscript, statistical analysis;

NE study conception and design, procurement of funding, critical revision of the manuscript for important intellectual content;

AST technical support and supervision, critical revision of the manuscript important for intellectual content;

DLW study conception and design, procurement of funding, critical revision of the manuscript for important intellectual content, supervision.
